# Potential Benefits and Risks Resulting From the Introduction of Health Apps and Wearables Into the German Statutory Health Care System: Scoping Review

**DOI:** 10.2196/16444

**Published:** 2020-09-23

**Authors:** Alexandra Heidel, Christian Hagist

**Affiliations:** 1 Chair of Economic and Social Policy WHU – Otto Beisheim School of Management Vallendar Germany

**Keywords:** health apps, wearables, digital health application, mHealth

## Abstract

**Background:**

Germany is the first country worldwide that has introduced a digital care act as an incentive system to enhance the use of digital health devices, namely health apps and wearables, among its population. The act allows physicians to prescribe statutory financed and previously certified health apps and wearables to patients. This initiative has the potential to improve treatment quality through better disease management and monitoring.

**Objective:**

The aim of this paper was to outline the key concepts related to the potential risks and benefits discussed in the current literature about health apps and wearables. Furthermore, this study aimed to answer the research question: Which risks and benefits may result from the implementation of the digital care act in Germany?

**Methods:**

We conducted the scoping study by searching the databases PubMed, Google Scholar, and JMIR using the keywords health apps and wearables. We discussed 55 of 136 identified articles published in the English language from 2015 to March 2019 in this paper using a qualitative thematic analysis approach.

**Results:**

We identified four key themes within the articles: Effectivity of health apps and wearables to improve health; users of health apps and wearables; the potential of bring-your-own, self-tracked data; and concerns and data privacy risks. Within these themes, we identified three main stages of benefits for the German health care system: Usage of health apps and wearables; continuing to use health apps and wearables; and sharing bring-your-own; self-tracked data with different agents in the health care sector.

**Conclusions:**

The digital care act could lead to an improvement in treatment quality through better patient monitoring, disease management, personalized therapy, and better health education. However, physicians should play an active role in recommending and supervising health app use to reach digital-illiterate or health-illiterate people. Age must not be an exclusion criterion. Yet, concerns about data privacy and security are very strong in Germany. Transparency about data processing should be provided at all times for continuing success of the digital care act in Germany.

## Introduction

Health apps and wearables have experienced increasing popularity in recent years [1]. Health apps and wearables are able to contribute more to the health care system than monitoring physical exercise, heart rate, or calories; they may support chronically ill patients with the management of specific diseases such as Parkinson’s disease, diabetes, tinnitus, or stress-related symptoms. Yet, Kotlikoff and Hagist [2] outlined already in 2009 that constantly increasing health care expenditure is one of the major social challenges for modern welfare states. Health apps and wearables might hold significant potential to decrease these costs. Apps and wearables are considered beneficial in the fields of preventive medicine and disease monitoring because the gamification of health enhances personal motivation and coordination. Germany has just launched one of the most progressive pilot projects in its health care history. The parliament passed the Digitale Versorgung Gesetz (DVG; digital care act) in 2019, which introduces the digitale Gesundheitsanwendungen (DIGA; digital health applications) into the German statutory health care system [3]. The DVG enables physicians to prescribe health apps for smartphones or wearables, which are covered for the insured by the sickness funds. This incentive system to introduce mobile health (mHealth) into the health care system is unique and exceptional worldwide [4]. The German Ministry of Health has shaped a completely new concept with the term DIGA. DIGA is a medical device within the scope of the European medical device regulation and classified as risk level I and not higher than a risk level IIa [5]. DIGA is a portable technology with the medical scope of monitoring, treatment, or reducing the effects of diseases [5]. Simple nutrition or menstrual cycle apps without any clear scope to improve the treatment effectivity of a medical condition are, for now, not considered as DIGAs.

Researchers in Germany are currently discussing the potential success of the act and the expected patient demand and acceptance. Experience with a regulation such as the DVG does not exist. According to a study by GfK, about 28% of Germans (25% female, 30% male) track at least one health parameter [6], and the average use from all 16 surveyed countries is 33%. Reasons to not track personal health data might be related to data security concerns, the accessibility of technology, or personal attitudes towards the recording of fitness parameters. We aimed to identify key concepts of the inclusion of health apps and wearables in the German statutory health care sector. We analyzed 55 of 136 identified articles to answer the research question: Which risks and benefits may result from the implementation of the digital care act in Germany?

## Methods

According to Munn et al [7], we conducted a scoping study to identify key concepts of the inclusion of health apps and wearables into the German statutory health care sector. The study aimed to draw a general picture about the risks and benefits of statutory financed mHealth solutions in Germany.

### Scoping Method

**Figure 1:**
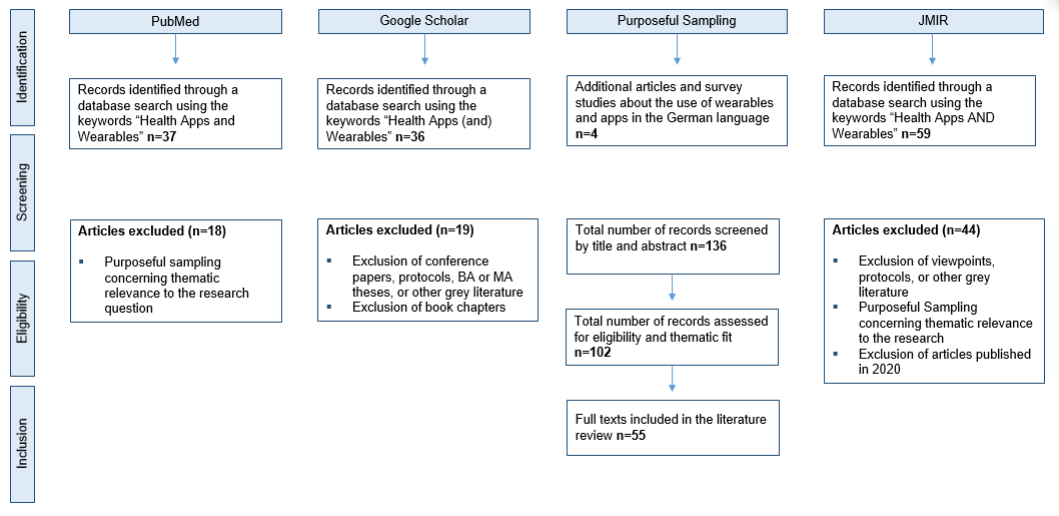
Scoping process for literature about health apps and wearables.

We performed this study according to the guidelines of scoping studies by Colquhoun et al [8]. Colquhoun et al [8] advanced the 6 stages of scoping studies by Arksey and O’Melley [9]. They elaborated on different stages of research such as the identification of a research question and literature, study selection, charting data, summarizing, and consulting [9]. To ensure rigor and transparency, this literature review was guided by our research question [9]. We started the scoping study with a database search of PubMed using the keywords “health apps AND wearables” (Figure 1). The scoping of literature was limited to articles published in the English language from 2015 to March 2019 because literature on health apps and wearables, as well as the boom of using those technologies, experienced a steep increase in 2015 [10]. The search identified 37 potential items. A second search was conducted via Google Scholar by using the keywords “health apps (and) wearables,” limiting the search again to literature published in the English language from 2015 to March 2019, and 36 items were identified. Then, another 2 articles in the German language and 2 survey studies in the German language were included in the study through purposeful sampling [11] because they were recommended. We conducted a third database search through JMIR using the search terms “health apps AND wearables” and identified 59 articles published from 2015 to March 2019 in English. We conducted other trial searches using other keywords such as “mHealth,” “fitness apps,” “health apps,” and “health data sharing” but the sampled literature had little fit with the research question. Hence, when searching only for the search term “health apps,” JMIR returned 698 search results. However, we chose the search term “health apps AND wearables” for our study because this is the closest that the published literature gets in terms of the German DIGA concept [12].

### Identification of Relevant Articles

Conference papers, conference reports, protocols, viewpoints, letters, Bachelor and Master theses, or other grey literature were not included. First, we screened articles by title and abstract. Literature relating to the themes of patient treatment with health apps or wearables, preventive care with apps or wearables, market studies about health app and wearable use, data privacy concerns, and patient use of health apps and wearables were included in this study. Hence, duplicates and ineligibility were further reasons for exclusion. Regarding the inclusion themes selected via purposeful sampling [11], 55 of 136 articles were included in this scoping study, and we analyzed the articles using a qualitative thematic analysis approach (see Multimedia Appendix 1 and Multimedia Appendix 2).

## Results

Of the 55 studies, 22 studies were literature, website, or app reviews; 16 studies were qualitative studies; and 17 studies were survey, interview, or quantitative studies. Most survey studies were not representative. Overall, we concluded that there is a growing amount of health app and wearable literature, but there is still room for additional research because not every aspect of the introduction of mHealth solutions into the health care system is known yet. There are few long-term studies on the effectivity of the use of health apps and wearables as a form of patient treatment. We have no insights about the effects of DIGA prescription and usage over 5, 10, or 20 years. Most articles we reviewed originated in Western Europe, the United States, and Canada.

After article scoping and conceptualization of the main findings, 4 main themes emerged: users of health apps and wearables; effectivity of health apps and wearables to improve health; the potential of bring-your-own, self-tracked data; and concerns and data privacy risks.

### Users of Health Apps and Wearables

A study by GfK reported that 33% of survey participants from 16 different countries used wearables or health apps to track their fitness or health on average [6]. The main reasons for people to use these devices is to improve their personal level of fitness or for self-motivation. In Germany, about 28% of people currently track their health — more men than women and rather younger than older people [6]. Another survey conducted by Statista [13] showed similar results. Users mainly focus on self-optimization. The youngest user group (18-29 years) has the largest proportion of app users [13].

Wiesner et al [14] conducted a field study and surveyed participants from a regional road race event about their use of wearables. They decided to survey sport-enthusiast runners because they anticipated that mainly young and active people use health apps and wearables. The study showed that 73% of the runner community used one or more wearables to track their activity [14]. Just 1% of the respondents used wearables sponsored by their health insurer [14]. The authors further asked about data privacy concerns of nonvoluntary data sharing, and 42% of the respondents “stated that they would not be concerned if data were shared in such a manner” [14]. This result might be significantly different when surveying a group of chronically ill or nonactive people. The results further show that the willingness to share data with different agents decreases for respondents in older age groups [14]. Most respondents of a US market study used health apps and wearables to monitor personal activity, nutrition, weight loss, or learn a new exercise [15]. The majority of the surveyed users used their health or fitness apps at least once a day [15]. Just 20% of the respondents discovered an app through the recommendation of a physician [15]. Among the most frequent reasons for people to not use health apps and wearables were lack of interest, high prices, and lack of trust in data security [15].

Park et al [16] conducted a similar study in South Korea and achieved similar results. The main reasons to use health apps and wearables were concerns about personal health status, self-optimization, innovative propensity, and trust in beneficial results. Surprisingly, the results indicated that the quality of the app has less influence on the decision whether to continue to use an app than social-cognitive factors [16]. Paré et al [17] also analyzed the motivation of people using health apps or wearables in Canada. They concluded that about 41% of the respondents used digital devices to self-track their health and physical activity (PA), which is significantly more than the German average. Furthermore, “a majority of digital self-trackers are young or mature adults (18-34 years), highly educated …, wealthy … and people who perceive themselves to be in good or very good health” [17]. Mosconi et al [18] and Ernsting et al [19] agreed with this statement and determined that young people in particular are interested in these technologies. Users feel generally more informed about their health when tracking different parameters, and 7 of 10 respondents improved or maintained their health condition by using an app or wearable [17]. Nevertheless, “one-third of consumer wearables end up in a drawer 6 months after purchase” [17]. This phenomenon occurs mainly with people with poor health or a chronic illness, indicating that this group loses interest in the technology when constantly reminded about a chronical condition or illness. Those people might feel pressured to be physically active [17].

Canhoto and Arp [20] agreed with Paré et al [17] by stating that many wearable and health app users stop using their devices after a while. Many insurance companies offer their members financial incentives and bonus programs to adopt a certain app or track specific health parameters [20]. The authors claimed that the inclusion of wearables and health apps in the health care system might have a significant positive influence on the treatment of chronic disease, like obesity or diabetes [20]. The widespread adoption and acceptance of these technologies are the key to their effectivity.

Christóvão [21] analyzed in his paper the influencing factors leading to app usage and the potential of health apps recommended and monitored by physicians. Perceived ease of use, perceived usefulness, peer influence, seniority, age, and gender were among the most important factors [21]. The author surveyed 199 fully qualified doctors and medical students to analyze the perceived usefulness of introducing health apps and wearables into patient care. Senior physicians and female physicians tended to use health apps less frequently if there was little peer influence, little perceived usefulness, and high complexity of usage [21]. A majority of the respondents could imagine recommending health apps and wearables to patients. Collado-Borrell et al [22], Davis et al [23], and Lipschitz et al [24] stated that many patients, nonetheless, already use health apps and wearables and are generally interested in the adaption of these technologies, independent of their age. However, Krebs and Duncan [15] rejected the view that all influencing factors are equally important. Wiesner et al [14] disagreed that gender significantly influences app usage, and Mackert et al [25] stated that health literacy plays an important role in the willingness to use these technologies.

Somers et al [26] conducted a contingent evaluation about the willingness-to-pay (WTP) for and willingness-to-accept (WTA) the use of health apps with different features. The results indicated that people value the promotion of wellbeing, social connectivity, and health care control [26]. Hence, Peng et al [27] identified the price of a wearable or health app as a significant influencing factor for the decision to adopt. The main reasons for people to abandon health apps or wearables after a certain period are, according to Peng et al [27], lack of time and effort and the lack of motivation and discipline. This means that apps or wearables alone cannot trigger a tremendous lifestyle change. The authors identified important reasons for people to use and continue to use health apps and wearables such as social competition, intangible rewards, tangible rewards, hedonic factors, and internal dedication [27]. To set incentives for nonactive or chronically ill patients to adopt health apps or wearables, tangible rewards like bonus programs might be the most promising tool in the future because “money is one of the biggest motivators” [27]. Petersen et al [28] concluded that tracking health parameters and communication through internet platforms triggers more self-consciousness and leads to patient empowerment.

### Effectivity of Health Apps and Wearables to Improve Health

A study from the German Ministry of Health [29] assigned health apps and wearables a significant role in the future and singled out the importance of incorporating self-tracked data into the physician’s daily routine and diagnostics. The stagnating telemedical development in Germany might be one of the major obstacles for the incorporation of DIGAs into the German health care system and needs further attention. However, Albrecht [29] argued that apps should be developed in cooperation with physicians, pharmaceutical companies, and health insurers to better meet the needs of the patients. The author claimed that the continuous use of health apps has a positive effect on personal health [29].

Mercer et al [30] conducted a participant’s study and provided wearables to 32 chronically ill participants (aged >50 years), which they evaluated according to questions derived from the technology acceptance model. They found out that older and chronically ill people perceive wearables as “useful and acceptable.” The use of wearables could enhance the level of PA because the devices create awareness of real motion [30]. Many older participants have not used a smartphone or tablet before and have strong concerns about their competencies. Yet, the technologies could remove barriers between physicians and patients [30]. Ehn et al [31] conducted a similar study. The authors concluded that the overall PA of the elderly increased during the study and that the wearables acted as a significant motivator [31]. However, they defined similar barriers for the use of wearables [31]. Schoeppe et al [32] reviewed 25 apps for children and adolescents and concluded that these apps have moderate quality overall. User engagement while using the app was not satisfactory, and the apps did not respond to individual needs. The authors ascribed to health apps for children and adolescents a high potential effectivity of sustainable behavioral change through gamification. They suggested, similar to Albrecht [29], cooperation of physicians, pharmaceutical companies, health insurers, and app developers [32]. Hartzler et al [33] and Hoffmann et al [34] also stressed the inclusion of gamification and interactive features as main factors for the success of health apps and wearables.

Firth and Torous [35] concluded their literature search by stating that there is still little empirical research available on the effectivity of health apps, specifically as a complementary treatment for schizophrenia: “People with schizophrenia are willing and able to use smartphones to monitor their symptoms, engage in self-directed therapeutic interventions, and increase their physical exercise.” Patients not officially diagnosed with schizophrenia or patients in acute stages report problems with app adherence [35]. Urrea et al [36] predicted that the use of health apps is an effective tool for the prevention of cardiovascular disease. Interventions via apps related to improvement and monitoring of smoking behavior, nutrition, and PA show positive results [36]. Hartmann et al [37], Christmann et al [38], and Ose et al [39] found significant potential of health apps and wearables for the treatment of depression. DIGAs might personalize care and reduce communication barriers with medical doctors. Gabriels and Moerenhout [40] and Martinez-Millana et al [41] concluded that the use of health apps and wearables help improve patients’ awareness and health education.

### The Potential of Bring-Your-Own, Self-Tracked Data

Haghi et al [42] ascribed to bring-your-own, self-tracked data an important role because of the predictions and simulations that could be achieved using big data: “The Internet of Things is a new concept, providing the possibility of health care monitoring using wearable devices.” Health monitoring could be done to a large extent autonomously, using sensors like motion trackers, vital signs, and gas detectors [42]. Dimitrov [43] identified 4 main strategies: descriptive analysis, prescriptive analysis, predictive analysis, and simulations. The author predicted potential future savings in the health care sector because most patients could monitor their health by themselves and upload their data to a medical Internet of Things. Data analysis could be achieved using big data and digital health advisors, which could decrease the number of necessary annual visits to physicians [43]. Turankhia and Kaiser [44] agreed with Dimitrov [43] and identified the monitoring of patients at risk of atrial fibrillation with health apps and wearables as tools to increase the rate of early detection and therefore decrease physician visits. Heintzman [45] also argued that the management and monitoring of diabetes through apps could decrease costs for the health care system because the technologies offer individualized guidance. Henriksen et al [10] criticized that self-tracked data is, in most cases, uploaded to brand-specific repositories, which makes it difficult to share data with medical staff or compare data between different applications.

Vahabzadeh et al [46] identified mHealth primarily as a game changer in the treatment of depression and even as a measure of suicide prevention. The author stated that there is great potential to detect the risk of suicide early and to help individuals with specific apps tailored to their needs. However, medical doctors should not solely rely on these technologies for detection and treatment, given the tremendous pitfalls of a potential error [46]. Lüttke et al [47] agreed with the points made by Vahabzadeh et al [46]. They see great potential in the use of DIGAs as complementary to therapy.

Genes et al [48] researched the effectivity of asthma monitoring through health apps and concluded that there was improvement in asthma control and a decrease in necessary physician contact. More importantly, the use of the app helped to reduce barriers within patient-physician communication [48]. Yet, another study showed that the incorporation of bring-your-own, self-tracked data in preventive care programs might be very promising. The reason for the positive outlook is the advancement of patient education through data visualization and a better self-monitoring strategy [1]. However, the widespread adoption of these technologies and integration of the data in routine physician care are challenging [1]. Lobelo et al [1] recommended that health app developers, researchers, regulators, and medical staff conjointly develop solutions to ensure compliance, compatibility, and health data security. Brandt et al [49] conducted a study by interviewing general practitioners in Denmark, and a majority of the general practitioners already used health apps and are generally convinced about the effectiveness but do not “translate that into lifestyle change guidance for their patients.” The authors suggested that health apps and wearables have significant potential to improve diagnostics and are a complimentary treatment for patients.

Chung et al [50] found that patients get better insights about their specific condition and feel empowered and connected. Cresswell et al [51] ascribed the integration of bring-your-own, self-tracked data into the daily routine of physicians as an aspirational role in preventive care and diagnostics. Furthermore, Cresswell et al [51] agreed with Chung et al [50] that self-monitoring of vital parameters and data visualization empower and educate patients.

Knight and Bidargaddi [52] concluded that self-management of mental diseases through apps leads to patient empowerment and the improvement of clinical care through better understanding. Ramkumar et al [53] agreed with this argument.

### Concerns and Data Privacy Risks

Wichmann et al [54] criticized, despite all the potential benefits, the general academic enthusiasm about introducing DIGAs into the health care system, even though there is little empirical evidence about their long-term effectivity, or the usage over several years. Urban [55] conducted qualitative interviews to research the user perception of elderly people. The author claimed that health apps and wearables motivate elderly people to increase their activity, but they also cause them “to develop negative emotions that stand in a charged relationship to aging stereotypes.” Elderly, who suffer from severe chronic conditions, feel discomfort integrating these technologies into their daily routine because the apps constantly remind them of their illness [55].

McCallum et al [56] agreed with Urban [55] and argued that the use of DIGAs are currently limited to mainly young and sportive people. To achieve widespread use, the usability and acceptability, especially of people with chronic conditions, need to be improved [56]. Data security issues are one of the main concerns for chronically ill people because they fear discrimination in different parts of their daily life [56]. Montgomery et al [57] supported this claim and demanded government regulation to enhance fairness and equity but also to protect personal data from the sale to third parties.

Groß and Schmidt [58] suggested that patients could be overstrained with the amount of data and sensors available. Hence, patients are not sufficiently trained to read and properly analyze health data and peak graphs. They are not able to assess the data and identify their relevance, which could lead to misinterpretation [58]. The authors also listed positive effects resulting from the use of health apps and wearables for patients like efficiency, control, goal orientation, and better organization [58]. Another major problem discussed in the paper is the concern about data security, the consequences of potential data theft, and data sales to third parties [58].

Hicks et al [59] and Huckvale et al [60] discussed in their studies privacy risks that could result from the use of fitness and health apps. Users of health and fitness apps rely on the ethical operation of app services and need to trust the apps they use [59,60]. However, app services, especially those offering free operation, mainly sell the collected data to third parties and hide these conditions in very long policy terms. The authors examined the privacy policies of 79 popular health apps and found that 89% of the apps communicate with online services and 90% also communicate with “one or more third-party services directly” [60]. The authors criticized that most health and fitness apps “rely mainly on self-declared compliance” [60]. Armstrong [61] came to the same conclusion with a similar study and suggested government regulation for health data processing. Tabi et al [62] and Jamaladin et al [63] also criticized the lack of clarity of conventional app stores and emphasized the need for professional health app stores and medical doctors’ recommendation to their patients. Becker et al [64] agreed with Huckvale et al [60] and Armstrong [61] because most health apps are not certified as a medical device, which means that their data protection terms are, in most cases, not supervised by a government agency. However, certification processes take a long time and are expensive. Incentives for the certification of apps are currently missing. However, the German digital care act enables fast track certification for DIGAs, which allows for early market access and a 1-year test phase to prove a positive health care effect [3].

## Discussion

During the analysis of 55 of the 136 papers, we found 4 main themes or concepts regarding the introduction of DIGAs in the health care system: users of health apps and wearables; effectivity of health apps and wearables; the potential of bring-your-own, self-tracked data; and concerns and data privacy risks. In terms of the introduction of the digital care act in Germany, health apps and wearables are supposed to have an overall positive effect for patients. The literature shows that patients with chronic conditions especially could benefit from the DVG through self-monitoring and health education but also through reduced communication barriers with their physicians [29-31,35,43].

However, there is still a lack of long-term empirical evidence about the effect of statutory financed DIGAs. Yet, it is not very clear how health app and wearable developers should prove a positive effect on medical care for patients after their 1-year test-phase. Long test phases and costly control group trials are not feasible for health apps and wearables [5]. Many authors criticize the pure amount of health apps and wearables available on the market and the difficulty for people to choose one specific to their needs. They argue that integrating health care staff into the process of app development and recommendation and supervision by physicians would increase the potential benefits of the technology [1,10].

There are not just potential benefits but also severe direct and indirect privacy concerns and the fear of discrimination, for example, through the employer or health insurance company [65]. Users, especially in Germany, lack trust in many app providers concerning their data because of missing transparency. This is the reason why data privacy and data security are a major part of the DIGA certification process resulting from the digital care act. Hence, this is also why patient-tracked data is not automatically forwarded to the statutory sickness funds or the physicians. The patient should remain the owner of his data [5].

Transparency about data processing might be one of the major solutions to data privacy concerns. Users are generally more willing to share their data if application services are transparent about data processing than if it remains unclear or the user feels betrayed [66]. In European countries, personal data is understood to be personal property, and regulations such as the European General Data Protection Regulation (DSGVO) are set to protect this property [66].

In a second digitization phase, Germany could introduce another regulation that enables health care providers to offer patients a digital dividend to use their self-tracked data for research purposes. However, to price self-tracked health data might be very difficult because the users generally overestimate the price of their personal data: “By its nature, personal data is non-rival, cheap to produce, cheap to copy, and cheap to transmit” [66].

A recent study showed that many people in Germany are willing to share personal data in exchange for benefits or rewards: 12% agreed, 40% disagreed, and 48% did not want to answer the question [67]. Yet, 30 million German consumers use the Payback program initiated by the American Express Group, which involves selling consumer data for bonus points in certain stores [68]. Many people are not directly aware of the fact that they sell their data to Payback GmbH and the company sells the data to third parties [69]. When directly asked, people are often very sensitive to the commercial exploitation of personal data [70]. In the experiment by Cvrcek et al [70], the median bid accepted for location data was €43 (US $51.06). An experiment by Grossklags and Acquisti [71] showed that most participants are willing to sell their data but are not willing to pay for the protection. The average WTA for their data about individual quiz performance was US $7.06 and for their personal personal information was US $31.80. The WTP to protect both types of data was US $0.80 [71]. The authors discovered that the type of personal data is individual and emotionally charged, influencing the WTP and WTA decision. When participants were asked about the number of their previous sexual partners, average WTA was US $2291.30, and WTP was US $12.10 [71]. Going a step further, when asked to auction their weight, age, and height, probands with a BMI below average demanded lower compensation to make their weight publicly available than probands with a BMI above average [72].

Hence, Von Wedel et al [73] showed that there is general interest in the inclusion of digital and mobile services in the German health care system. Yet, this gives a positive outlook for the success of the digital care act in Germany. According to the studies reviewed, we predict a high demand for DIGAs from young and healthy adults in the beginning. Yet, we believe that chronically ill and elderly patients benefit to a large extent from the regulation, which is why physicians and doctors should act as mediators and recommend, supervise, and accompany app use.

**Figure 2:**
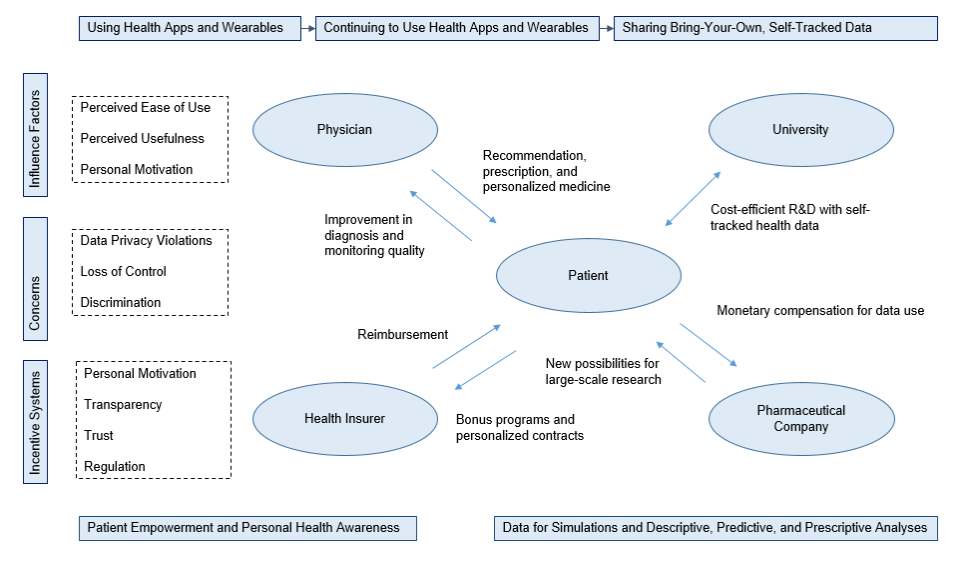
Stages from use to continuous use to the sharing of self-tracked data.

Three main stages of potential benefits for the German health care system were identified in the literature: usage of health apps and wearables [14,17,46], continuing usage of health apps and wearables [36,55], and sharing self-tracked data with agents in the health care sector [42,48]. Figure 2 shows the different stages mapped against the identified influence factors, concerns, and potential incentive systems.

The literature assigns each of the stages potential benefits when integrated into the health care system. The decision if individuals use health apps depends to a large extent on the perceived ease of use, perceived usefulness, trust, peer influence, personal health status, and technology literacy. Main concerns about the use of health apps and wearables discussed are data privacy violations or physical discomfort [15,16]. Whether an individual decides to continue to use a health app or wearable depends on the usefulness of the app to achieve certain goals, personal discipline, motivation, and trust. The concerns about continuing to use an app or wearable seem to be almost identical to the ones about starting to use an app, but even more sensitive to personal discomfort and the individual distortions of chronic diseases [21,27]. Presuming that the use of health apps and wearables has positive effects on the prevention of certain disease or aids treatments, the reasons why people stop using apps should be further studied, as well as potential incentive systems to assist people to continue to use these apps.

Some incentives named within the literature are bonus programs or physicians’ recommendations. The last stage is the potential and willingness to bring along or share self-tracked data with different agents in the health care system. People seem to have very strong concerns about voluntarily sharing their self-tracked health data, which range from price discriminations to a lack of transparency and social embarrassment [26].

Referring to the research question of this paper, the digital care act and the introduction of statutory financed DIGAs could be considered societally beneficial. The widespread use of DIGAs allows patient empowerment, better monitoring of chronic diseases, and individualized advice. These benefits could not only reduce the number of mandatory visits to physicians and therefore the evergrowing expenses for the health care system but also lead to better resource allocation and improved treatment quality. Yet, Germany is the first country worldwide to introduce prescribed DIGAs. This is a significant chance to enhance digitization in the German health care sector and to build a foundation for a digital dividend to buy self-tracked patient data for research purposes. Yet, this experiment also bears risks when considering the volatile patient trust in data security.

### Limitations

This study might be affected by the limited amount of available research resulting from the search terms. This might give a unilateral perspective on the effectivity of health apps and wearables. Hence, we are always concerned about the selection bias of articles. However, the multidisciplinary perspective on the field of study, enhanced through articles from different schools of thought and different research disciplines, as well as the applied rigor of scoping studies, have contributed to eliminate the selection bias to a large extent. Further research should be conducted after the first DIGAs are certified and have entered the German health care market.

### Conclusions

To conclude, 55 of the 136 articles were analyzed within this scoping study. First, 4 key themes were identified: users of health apps and wearables; effectivity of health apps and wearables to improve health; potential of bring-your-own, self-tracked data; and concerns and data privacy risks.

In December 2019, Germany passed the digital care act, which enables the statutory financed prescription of digital health devices by medical doctors. Based on this scoping study, we predict an overall beneficial effect for German patients, treatment quality, and general health literacy of the population. The main benefits are going to be visible in the fields of preventive care and patient monitoring and disease management. Three main stages of potential benefits for the health care system were identified: using health apps and wearables, continuing to use health apps and wearables, and sharing bring-your-own, self-tracked data with different agents in the health care sector.

## Appendix

Multimedia Appendix 1 Study characteristics.

Multimedia Appendix 2 Study aims and findings.

## Abbreviations

DIGA: digitale Gesundheitsanwendungen
DVG: Digitale Versorgung Gesetz
mHealth: mobile health
WTA: willingness-to-accept
WTP: willingness-to-pay

## References

[1] Lobelo F, Kelli HM, Tejedor SC, Pratt M, McConnell MV, Martin SS, Welk GJ (2016). The Wild Wild West: A Framework to Integrate mHealth Software Applications and Wearables to Support Physical Activity Assessment, Counseling and Interventions for Cardiovascular Disease Risk Reduction. Prog Cardiovasc Dis.

[2] Kotlikoff L, Hagist C (2005). Who's Going Broke? Comparing Growth in Healthcare Costs in Ten OECD Countries. NBER Working Paper No. w11833.

[3] Der Bundestag (2019). Bundesgesetzblatt Teil I Nr. 49. Gesetz für eine bessere Versorgung durch Digitalisierung und Innovation (Digitale Versorgung Gesetz).

[4] Zeit Online (2019). Jens Spahn: Krankenkassen Sollen Für Gesundheitsapps Zahlen. Die Zeit.

[5] Bundesinstitut für Arzneimittel und Medizinprodukte (2020). Das Fast-Track-Verfahren Für Digitale Gesundheitsanwendungen (DiGA) Nach § 139e SGB V. DIGA Leitfaden.

[6] GfK (2016). Health and Fitness Tracking: Global GfK Survey.

[7] Munn Z, Peters MDJ, Stern C, Tufanaru C, McArthur A, Aromataris E (2018). Systematic review or scoping review? Guidance for authors when choosing between a systematic or scoping review approach. BMC Med Res Methodol.

[8] Colquhoun HL, Levac D, O'Brien KK, Straus S, Tricco AC, Perrier L, Kastner M, Moher D (2014). Scoping reviews: time for clarity in definition, methods, and reporting. J Clin Epidemiol.

[9] Arksey H, O'Malley L (2005). Scoping studies: towards a methodological framework. International Journal of Social Research Methodology.

[10] Henriksen A, Haugen Mikalsen M, Woldaregay AZ, Muzny M, Hartvigsen G, Hopstock LA, Grimsgaard S (2018). Using Fitness Trackers and Smartwatches to Measure Physical Activity in Research: Analysis of Consumer Wrist-Worn Wearables. J Med Internet Res.

[11] Palinkas LA, Horwitz SM, Green CA, Wisdom JP, Duan N, Hoagwood K (2015). Purposeful Sampling for Qualitative Data Collection and Analysis in Mixed Method Implementation Research. Adm Policy Ment Health.

[12] Bundesministerium für Gesundheit (2020). Verordnung über das Verfahren und die Anforderungen der Prüfung der Erstattungsfähigkeit digitaler Gesundheitsanwendungen in der gesetzlichen Krankenversicherung, 2020. Bundesgesetzblatt Teil I Nr. 18.

[13] Radtke R (2018). Umfrage Zur Teilungsbereitschaft Von Gesundheitsdaten in Deutschland, 2016. Statista.

[14] Wiesner M, Zowalla R, Suleder J, Westers M, Pobiruchin M (2018). Technology Adoption, Motivational Aspects, and Privacy Concerns of Wearables in the German Running Community: Field Study. JMIR Mhealth Uhealth.

[15] Krebs P, Duncan DT (2015). Health App Use Among US Mobile Phone Owners: A National Survey. JMIR Mhealth Uhealth.

[16] Park M, Yoo H, Kim J, Lee J (2018). Why do young people use fitness apps? Cognitive characteristics and app quality. Electron Commer Res.

[17] Paré G, Leaver C, Bourget C (2018). Diffusion of the Digital Health Self-Tracking Movement in Canada: Results of a National Survey. J Med Internet Res.

[18] Mosconi P, Radrezza S, Lettieri E, Santoro E (2019). Use of Health Apps and Wearable Devices: Survey Among Italian Associations for Patient Advocacy. JMIR Mhealth Uhealth.

[19] Ernsting C, Stühmann LM, Dombrowski SU, Voigt-Antons J, Kuhlmey A, Gellert P (2019). Associations of Health App Use and Perceived Effectiveness in People With Cardiovascular Diseases and Diabetes: Population-Based Survey. JMIR Mhealth Uhealth.

[20] Canhoto AI, Arp S (2016). Exploring the factors that support adoption and sustained use of health and fitness wearables. Journal of Marketing Management.

[21] Veríssimo JMC (2018). Usage intensity of mobile medical apps: A tale of two methods. Journal of Business Research.

[22] Collado-Borrell R, Escudero-Vilaplana V, Calles A, Garcia-Martin E, Marzal-Alfaro B, Gonzalez-Haba E, Herranz-Alonso A, Sanjurjo-Saez M (2018). Oncology Patient Interest in the Use of New Technologies to Manage Their Disease: Cross-Sectional Survey. J Med Internet Res.

[23] Davis TL, DiClemente R, Prietula M (2016). Taking mHealth Forward: Examining the Core Characteristics. JMIR Mhealth Uhealth.

[24] Lipschitz J, Miller CJ, Hogan TP, Burdick KE, Lippin-Foster R, Simon SR, Burgess J (2019). Adoption of Mobile Apps for Depression and Anxiety: Cross-Sectional Survey Study on Patient Interest and Barriers to Engagement. JMIR Ment Health.

[25] Mackert M, Mabry-Flynn A, Champlin S, Donovan EE, Pounders K (2016). Health Literacy and Health Information Technology Adoption: The Potential for a New Digital Divide. J Med Internet Res.

[26] Somers C, Grieve E, Lennon M, Bouamrane M, Mair FS, McIntosh E (2019). Valuing Mobile Health: An Open-Ended Contingent Valuation Survey of a National Digital Health Program. JMIR Mhealth Uhealth.

[27] Peng W, Kanthawala S, Yuan S, Hussain SA (2016). A qualitative study of user perceptions of mobile health apps. BMC Public Health.

[28] Petersen A, Schermuly AC, Anderson A (2019). The shifting politics of patient activism: From bio-sociality to bio-digital citizenship. Health (London).

[29] Albrecht UV (2016). Opportunities and risks of health apps (CHARISMHA).

[30] Mercer K, Giangregorio L, Schneider E, Chilana P, Li M, Grindrod K (2016). Acceptance of Commercially Available Wearable Activity Trackers Among Adults Aged Over 50 and With Chronic Illness: A Mixed-Methods Evaluation. JMIR Mhealth Uhealth.

[31] Ehn M, Eriksson LC, Åkerberg N, Johansson A (2018). Activity Monitors as Support for Older Persons' Physical Activity in Daily Life: Qualitative Study of the Users' Experiences. JMIR Mhealth Uhealth.

[32] Schoeppe S, Alley S, Rebar AL, Hayman M, Bray NA, Van Lippevelde W, Gnam J, Bachert P, Direito A, Vandelanotte C (2017). Apps to improve diet, physical activity and sedentary behaviour in children and adolescents: a review of quality, features and behaviour change techniques. Int J Behav Nutr Phys Act.

[33] Hartzler AL, BlueSpruce J, Catz SL, McClure JB (2016). Prioritizing the mHealth Design Space: A Mixed-Methods Analysis of Smokers' Perspectives. JMIR Mhealth Uhealth.

[34] Hoffmann A, Christmann CA, Bleser G (2017). Gamification in Stress Management Apps: A Critical App Review. JMIR Serious Games.

[35] Firth J, Torous J (2015). Smartphone Apps for Schizophrenia: A Systematic Review. JMIR Mhealth Uhealth.

[36] Urrea B, Misra S, Plante TB, Kelli HM, Misra S, Blaha MJ, Martin SS (2015). Mobile Health Initiatives to Improve Outcomes in Primary Prevention of Cardiovascular Disease. Curr Treat Options Cardiovasc Med.

[37] Christmann CA, Hoffmann A, Bleser G (2017). Stress Management Apps With Regard to Emotion-Focused Coping and Behavior Change Techniques: A Content Analysis. JMIR Mhealth Uhealth.

[38] Hartmann R, Sander C, Lorenz N, Böttger D, Hegerl U (2019). Utilization of Patient-Generated Data Collected Through Mobile Devices: Insights From a Survey on Attitudes Toward Mobile Self-Monitoring and Self-Management Apps for Depression. JMIR Ment Health.

[39] Ose SO, Færevik H, Kaasbøll J, Lindgren M, Thaulow K, Antonsen S, Burkeland O (2019). Exploring the Potential for Use of Virtual Reality Technology in the Treatment of Severe Mental Illness Among Adults in Mid-Norway: Collaborative Research Between Clinicians and Researchers. JMIR Form Res.

[40] Gabriels K, Moerenhout T (2018). Exploring Entertainment Medicine and Professionalization of Self-Care: Interview Study Among Doctors on the Potential Effects of Digital Self-Tracking. J Med Internet Res.

[41] Martinez-Millana A, Jarones E, Fernandez-Llatas C, Hartvigsen G, Traver V (2018). App Features for Type 1 Diabetes Support and Patient Empowerment: Systematic Literature Review and Benchmark Comparison. JMIR Mhealth Uhealth.

[42] Haghi M, Thurow K, Stoll R (2017). Wearable Devices in Medical Internet of Things: Scientific Research and Commercially Available Devices. Healthc Inform Res.

[43] Dimitrov DV (2016). Medical Internet of Things and Big Data in Healthcare. Healthc Inform Res.

[44] Turakhia MP, Kaiser DW (2016). Transforming the care of atrial fibrillation with mobile health. J Interv Card Electrophysiol.

[45] Heintzman ND (2015). A Digital Ecosystem of Diabetes Data and Technology: Services, Systems, and Tools Enabled by Wearables, Sensors, and Apps. J Diabetes Sci Technol.

[46] Vahabzadeh A, Sahin N, Kalali A (2016). Digital Suicide Prevention: Can Technology Become a Game-changer?. Innov Clin Neurosci.

[47] Lüttke S, Hautzinger M, Fuhr K (2018). [E-Health in diagnosis and therapy of mental disorders : Will therapists soon become superfluous?]. Bundesgesundheitsblatt Gesundheitsforschung Gesundheitsschutz.

[48] Genes N, Violante S, Cetrangol C, Rogers L, Schadt EE, Chan YY (2018). From smartphone to EHR: a case report on integrating patient-generated health data. NPJ Digit Med.

[49] Brandt CJ, Søgaard GI, Clemensen J, Sndergaard J, Nielsen JB (2018). General Practitioners' Perspective on eHealth and Lifestyle Change: Qualitative Interview Study. JMIR Mhealth Uhealth.

[50] Chung AE, Sandler RS, Long MD, Ahrens S, Burris JL, Martin CF, Anton K, Robb A, Caruso TP, Jaeger EL, Chen W, Clark M, Myers K, Dobes A, Kappelman MD (2016). Harnessing person-generated health data to accelerate patient-centered outcomes research: the Crohn's and Colitis Foundation of America PCORnet Patient Powered Research Network (CCFA Partners). J Am Med Inform Assoc.

[51] Cresswell KM, McKinstry B, Wolters M, Shah A, Sheikh A (2019). Five key strategic priorities of integrating patient generated health data into United Kingdom electronic health records. J Innov Health Inform.

[52] Knight A, Bidargaddi N (2018). Commonly available activity tracker apps and wearables as a mental health outcome indicator: A prospective observational cohort study among young adults with psychological distress. J Affect Disord.

[53] Ramkumar PN, Muschler GF, Spindler KP, Harris JD, McCulloch PC, Mont MA (2017). Open mHealth Architecture: A Primer for Tomorrow's Orthopedic Surgeon and Introduction to Its Use in Lower Extremity Arthroplasty. J Arthroplasty.

[54] Wichmann F, Sill J, Hassenstein MJ, Zeeb H, Pischke CR (2018). Apps zur Förderung von körperlicher Aktivität. Präv Gesundheitsf.

[55] Urban M (2017). 'This really takes it out of you!' The senses and emotions in digital health practices of the elderly. Digit Health.

[56] McCallum C, Rooksby J, Gray CM (2018). Evaluating the Impact of Physical Activity Apps and Wearables: Interdisciplinary Review. JMIR Mhealth Uhealth.

[57] Montgomery K, Chester J, Kopp K (2018). Health Wearables: Ensuring Fairness, Preventing Discrimination, and Promoting Equity in an Emerging Internet-of-Things Environment. Journal of Information Policy.

[58] Groß D, Schmidt M (2018). [Ethical perspectives on E‑health and health apps : Is all that is achievable desirable?]. Bundesgesundheitsblatt Gesundheitsforschung Gesundheitsschutz.

[59] Hicks JL, Althoff T, Sosic R, Kuhar P, Bostjancic B, King AC, Leskovec J, Delp SL (2019). Best practices for analyzing large-scale health data from wearables and smartphone apps. NPJ Digit Med.

[60] Huckvale K, Prieto JT, Tilney M, Benghozi P, Car J (2015). Unaddressed privacy risks in accredited health and wellness apps: a cross-sectional systematic assessment. BMC Med.

[61] Armstrong S (2016). What happens to data gathered by health and wellness apps?. BMJ.

[62] Jamaladin H, van de Belt TH, Luijpers LC, de Graaff FR, Bredie SJ, Roeleveld N, van Gelder MM (2018). Mobile Apps for Blood Pressure Monitoring: Systematic Search in App Stores and Content Analysis. JMIR Mhealth Uhealth.

[63] Tabi K, Randhawa AS, Choi F, Mithani Z, Albers F, Schnieder M, Nikoo M, Vigo D, Jang K, Demlova R, Krausz M (2019). Mobile Apps for Medication Management: Review and Analysis. JMIR Mhealth Uhealth.

[64] Becker S, Miron-Shatz T, Schumacher N, Krocza J, Diamantidis C, Albrecht U (2014). mHealth 2.0: Experiences, Possibilities, and Perspectives. JMIR Mhealth Uhealth.

[65] Wathieu L, Friedman AA (2007). An Empirical Approach to Understanding Privacy Valuation. SSRN Journal.

[66] Spiekermann S, Acquisti A, Böhme R, Hui K (2015). The challenges of personal data markets and privacy. Electron Markets.

[67] GfK (2017). Global GfK Survey: Willingness to Share Personal Data in Exchange for Benefits or Rewards.

[68] Payback G (2019). Daten & Fakten. Payback GmbH.

[69] Biermann K (2019). Payback zahlt nichts zurück. Die Zeit.

[70] Cvrcek D, Kumpost M, Matyas V, Danezis G (2006). A Study on the Value of Location Privacy.

[71] Acquisti A, Grossklags J (2007). Privacy Attitudes and Privacy Behavior: Losses, Gains, and Hyperbolic Discounting. Economics of Information Security.

[72] Huberman B, Adar E, Fine L (2005). Valuating Privacy. IEEE Secur. Privacy Mag.

[73] von Wedel P, Hagist C, Saunders K (2018). Erratum: Die Digitalisierung der Arzt-Patienten Beziehung in Deutschland: Ein Discrete Choice Experiment zur Analyse der Patientenpräferenzen bezüglich digitaler Gesundheitsleistungen. Gesundh ökon Qual manag.

