# Supplementary material for: Potential Benefits and Risks Resulting From the Introduction of Health Apps and Wearables Into the German Statutory Health Care System: Scoping Review
Source: JMIR Mhealth Uhealth. 2020 Sep 23;8(9):e16444. doi: 10.2196/16444 (PMC7542416; doi:10.2196/16444)
Supplement: Multimedia Appendix 1 [file mhealth_v8i9e16444_app1.docx]

Multimedia Appendix 1. Study Characteristics

| # | Study | Method | Data Source | Data Type | Sample Size | Sample Country |
| --- | --- | --- | --- | --- | --- | --- |
| PubMed Search: Health Apps and Wearables n= 37, Eligible n= 20 | | | | | | |
| 1 | Henriksen et al (2018): "Using Fitness Trackers and Smartwatches to Measure Physical Activity in Research" | Literature Review | Six Databases (Queen's University Wearable Device Inventory, Gsm Arena, Wearables.com, SpecBucket, PrisGuide, the Vandrico Wearable Database) | Wearable Devices | 423 wearables from 132 different brands | n.a. |
| 2 | Mercer et al (2016): "Acceptance of commercially available wearable activity trackers among adults aged over 50 and with chronic illness" | Qualitative Mixed Methods Approach, | Participants study testing four different health trackers | Health data from participants and user perception | 30 chronically ill participants aged between 52 and 85 years | Canada |
| 3 | Firth & Torous (2015): "Smartphone Apps for Schizophrenia: A Systematic Review" | Literature Review | seven Databases (Ovid MEDLINE, the Cochrane Central Register of Controlled Trials, Health Technology Assessment Database, Allied and Complementary Medicine, Health and Psychosocial Instruments, PsycINFO, and Embase | Literature | 226 Articles in total, 7 eligible articles, 5 studies for Apps AND Schizophrenia | n.a. |
| 4 | Dimitrov (2016): "Medical Internet of Things and Big Data in Healthcare" | Descriptive Qualitative Study | Selected Articles | Literature | 27 Articles | n.a. |
| 5 | Haghi et al (2017): "Wearable Devices in Medical Internet of Things: Scientific Research and Commercially Available Devices" | Literature Review | Selected Articles | Literature | 51 Articles | n.a. |
| 6 | Vahabzadeh (2016): "Digital Suicide Prevention: Can Technology Become a Game-changer?" | Literature Review | Selected Articles | Literature | 17 Articles | USA |
| 7 | Lobelo et al (2016): "The wild wild west: A Framework to integrate mHealth software applications and wearables to support physical activity assessment, counseling and interventions for Cardiovascular Disease risk reduction" | Literature Review | MEDLINE, PubMed, Web of Science, World Wide Web | Literature | 95 Articles | n.a. |
| 8 | Wiesner et al (2018): "Technology Adaption, Motivational Aspects, and Privacy Concerns of Wearables in the German running community: Field Study" | Field Study, Survey | People at a regional road race event in 2017 | Questionnaires | 845 Surveys of runners | Germany |
| 9 | Urban (2017): "This really takes it out of you! The senses and emotions in digital health practices of the elderly" | Qualitative Case Study | Elderly people | Semi-Structured Interviews | 27 Middle-class elderly male and female people | Germany |
| 10 | Ehn et al (2018): "Activity Monitors as support for older persons' physical activity in daily life: Qualitative Study of Users' Experience" | Qualitative Study | Elderly people (median age: 83 years) | Interviews and User Diaries | 8 Community-dwelling elderly people (median age: 83 years) testing two different wearable and tablet based Apps for 74 test days | Sweden |
| 11 | Brandt et al (2018): "General Practitioners' Perspective on eHealth and lifestyle change: qualitative interview study" | Qualitative Study | General practitioners from Southern Denmark (Purposeful sampled) | Semi-Structured Interviews | 10 General practitioners (5 male, 5 female), engaged in electronic consultations | Denmark |
| 12 | McCallum et al (2018): "Evaluating the Impact of Physical Activity Apps and Wearables: Interdisciplinary Review" | Literature Review | 8 data bases (health and computing science) | Literature | 111 Articles on physical activity and its measurement through Apps | n.a. |
| 13 | Cresswell et al (2018): "Five key strategic priorities of integrating patient generated health data into United Kingdom electronic health records" | Qualitative Study | Literature from the American Medical Informatics Association | Literature | n.a. | North America, UK |
| 14 | Paré et al (2018): "Diffusion of the Digital Health Self-Tracking Movement in Canada: Results of a National Survey" | National Survey | Canadian adults | Questionnaires | 4109 Participants | Canada |
| 15 | Hicks et al (2019) “Best practices for analyzing large-scale health data from wearables and smartphone apps” | Review and report about quantitative PGHD Studies | Articles which use large-scale PGHD data sets | Literature and Data such as Step count | 75 Articles | n.a. |
| 16 | Lüttke et al (2018): “E-Health in diagnosis and therapy of mental disorders. Will therapists soon become superfluous?” | Literature Review | Articles on mHealth solutions for mental disease | Literature | 30 Articles | n.a. |
| 17 | Turankhia & Kaiser (2016): “Transforming the care of atrial fibrillation with mobile health” | Literature Review | Articles on mHealth tracking of AF patients | Literature | 31 Articles | n.a. |
| 18 | Heintzman (2016). “A Digital Ecosystem of Diabetes Data and Technology: Services, Systems, and Tools Enabled by Wearables, Sensors, and Apps” | Literature Review | Articles on mHealth Solutions to monitor and manage Diabetes | Literature | 97 Articles | n.a. |
| 19 | Knight & Bidargaddi (2018): ” Commonly available activity tracker apps and wearables as a mental health outcome indicator: A prospective observational cohort study among young adults with psychological distress” | Prospective Observational Cohort Trial Study | Mental Health Website ResearchOut.com | Self-reports on mental health and PGHD from other apps | 120 male and female participants between 18-25 years old | Australia |
| 20 | Ramkumar et al (2017): “Open mHealth Architecture: A Primer for Tomorrow’s Orthopedic Surgeon and Introduction to its use in Lower Extremity Arthroplasty” | Review | Articles about mHealth and surgery | Literature | 38 | n.a. |
| Google Scholar Search: "Health Apps (and) Wearables" n= 36 , Eligible n= 16, n= 4 Purposeful Sampled Articles in German | | | | | | |
| 21 | Canhoto & Arp (2017): "Exploring the factors that support adoption and sustained use of health and fitness wearables" | Exploratory Qualitative Study | Literature on consumer technology adoption and Members of fitness centers | Literature and Interviews | 20 participants in focus groups | Germany |
| 22 | Albrecht (2016): "Gesundheits-Apps und Prävention" | Literature Review | Pub Med. Scopus | Academic Articles | 86 Articles | n.a. |
| 23 | Groß & Schmidt (2018): "Health und Gesundheitsapps aus medizinischer Sicht" | Normative Ethical Analysis | Selected Articles | Theoretical Literature | n.a. | n.a. |
| 24 | Genes et al (2018): "From smartphone to EHR: a case report on integrating patient-generated health data" | Case Study | Asthma Patients | Self-tracked peak expiratory flow rate | 4 voluntary patients with asthma | USA |
| 25 | GfK (2016): "Global GfK survey: Health and Fitness Tracking" | Survey Study | Consumers answering the online Survey | Questionnaire | More than 20 000 consumers aged 15 and older | 16 Countries |
| 26 | Krebs & Duncan (2015): "Health App Use Among US Mobile Phone Owners: A National Survey" | National Survey | Cross-Sectional Survey | Questionnaire | 1604 Mobile Phone Users | USA |
| 27 | Becker et al (2014): "mHealth 2.0: Experiences, Possibilities, and Perspectives" | Qualitative Study | Various Databases | Literature | 67 Articles | n.a. |
| 28 | Huckvale et al (2015): "Unaddressed privacy risks in accredited health and wellness apps: a cross-sectional systematic assessment" | Cross-Sectional Systematic Assessment of Data Privacy Practices | Health App | In-App Data Privacy Policy | 79 Apps Certified as Clinically Safe | n.a. |
| 29 | Urrea et al (2015): "Mobile Health Initiatives to improve outcomes in primary prevention of cardiovascular disease" | Qualitative Thematic Study | Selected Articles | Literature | 50 Articles | n.a. |
| 30 | Statista (2018): "Mobile Health" | Survey Study | People living in Germany | Survey | 5046 Participants within the age group 16-69 years old | Germany |
| 31 | Park et al (2018): "Why do young people use fitness apps? Cognitive characteristics and App quality" | Interviews | College students | Self-report, Questionnaires | 201 Participants from three different Universities using at least one fitness App | South Korea |
| 32 | Schoeppe et al (2017): "Apps to improve diet, physical activity and sedentary behavior in children and adolescents: a review of quality, features and behavior change techniques" | Systematic Review of Health Apps for children | iTunes Store and Google Play Store | Health Apps rated at least with +4 based on 20 ratings, available in English, targeting children | 25 Apps | Australia |
| 33 | Wichmann et al (2018): "Apps for physical activity promotion. Attitudes, acceptance and utilization preferences among adults aged 50 years and above: results of focus group discussions" | Interview Study | Participants | Interviews and Discussions with Participants | 15 People in three focus groups older than 50 years | Germany |
| 34 | Cristóvao Veríssimo (2018): "Usage intensity of mobile medical apps: A tale of two methods" | Mixed Methods Approach; Logistic Regression and fsQCA | Fully qualified doctors and medical Students engaged in a Medical Social Network | Survey | 199 Respondents | USA |
| 35 | Peng et al (2016): "A qualitative study of user perceptions of mobile health apps" | Thematic Inductive Analysis | Purposive sampled Smartphone Users | Five individual interviews | 44 Participants | USA |
| 36 | Somers et al (2019): "Valuing Mobile Health: An Open-Ended Contingent Valuation Survey of a National Digital Health Program" | Contingent Valuation Willingness to Pay Study | ResearchNow panel Members | National Survey | 1679 Individuals and 305 Representatives of Dallas Intervention Communities | UK |
| 37 | Chung et al (2016): "Harnessing person-generated health data to accelerate patient-centered outcomes research: the Crohn’s and ColitisFoundation of America PCORnet Patient Powered Research Network" | Project Study to establish Patient centered Data Research | Clinical Data Research Network | Patient reports, outcomes and health behavior | 14200 IBD (inflammatory bowel disease) patients | USA |
| 38 | Petersen et al (2019): “The shifting politics of patient activism: From bio-sociality to bio-digital citizenship” | Media Review | Scopus, Medline, Google, University Library | Media produced by patient activist 1980- today | 76 Articles | n.a. |
| 39 | Armstrong (2016): “What happens to data gathered by health and wellness apps?” | Report | Articles on PGHD use | Literature | 18 Articles | n.a. |
| 40 | Montgomery et al (2018): “Health Wearables: Ensuring Fairness, Preventing Discrimination, and promoting Equity in an Emerging Internet-of-Things Environment” | Review | Articles on mHealth and Big Data | Literature | n.a. | n.a. |
| JMIR Publications Search: "Health Apps AND Wearables" n= 59 , Eligible n= 15 | | | | | | |
| 41 | Hartmann et al (2019): “Utilization of Patient-Generated Data Collected Through Mobile Devices: Insights From a Survey on Attitudes Toward Mobile Self-Monitoring and Self-Management Apps for Depression” | Online Survey | Online Panel Questback GmbH. | Questionnaires | 825 Participants | Germany |
| 42 | Mosconi et al (2019): “Use of Health Apps and Wearable Devices: Survey Among Italian Associations for Patient Advocacy” | Online Survey | Patients’ Health Care Advocacy Associations | Questionnaires | 227 completed Questionnaires | n.a. |
| 43 | Ernsting et al (2019): “Associations of Health App Use and Perceived Effectiveness in People With Cardiovascular Diseases and Diabetes: Population-Based Survey” | Online Survey | Online Panel Gesellschaft für Innovative Marktforschung | Questionnaires | 1500 Diabetes Patients | Germany |
| 44 | Gabriels & Moerenhout (2018): “Exploring Entertainment Medicine and Professionalization of Self-Care: Interview Study Among Doctors on the Potential Effects of Digital Self-Tracking” | Interview Study | Purposeful Sampling | Semistructured Interviews | 12 General Practitioners and Cardiologists | Belgium |
| 45 | Tabi et al (2019): “Mobile Apps for Medication Management: Review and Analysis” | Systematic App Search | iOS Apple App Store and Android Google Play Store | App Content | 328 Apps | n.a. |
| 46 | Jamaladin et al (2018): “Mobile Apps for Blood Pressure Monitoring: Systematic Search in App Stores and Content Analysis” | Systematic App Search | iOS Apple App Store and Android Google Play Store | Quality Scores | 184 Apps | n.a. |
| 47 | Christmann et al (2017): “Stress Management Apps With Regard to Emotion-Focused Coping and Behavior Change Techniques: A Content Analysis” | Content Analysis | Google Play Store | App Content | 62 Free Apps | n.a. |
| 48 | Collado-Borrell et al (2018): “Oncology Patient Interest in the Use of New Technologies to Manage Their Disease: Cross-Sectional Survey” | Cross-Sectional Survey | Hematology-Oncology Patients in a Day Hospital or the Pharmaceutical Care Consultancy | Questionnaires | 611 Patients | Spain |
| 49 | Morrow Lipschitz et al (2019): “Adoption of Mobile Apps for Depression and Anxiety: Cross-Sectional Survey Study on Patient Interest and Barriers to Engagement” | Cross-Sectional Survey Study | A single Veterans Health Administration | Questionnaires | 149 Veterans | USA |
| 50 | Martinez-Millana et al (2018): “App Features for Type 1 Diabetes Support and Patient Empowerment: Systematic Literature Review and Benchmark Comparison” | Systematic Literature Review and Benchmark Comparison | PubMed, Medline, Google Scholar, and Cochrane Trials | Literature | 55 Articles | n.a. |
| 51 | Mackert et al (2016): “Health Literacy and Health Information Technology Adoption: The Potential for a New Digital Divide” | Cross-Tabulation Analysis | Newest Vital Sign Measure of Health Literacy | Questionnaires | 4974 Adults | USA |
| 52 | Hoffmann et al (2017): “Gamification in Stress Management Apps: A Critical App Review | Review | Google Play Store | App Content | 62 Stress Management Apps | n.a. |
| 53 | Hartzler et al (2016): “Prioritizing the mHealth Design Space: A Mixed-Methods Analysis of Smokers’ Perspectives” | Mixed-Methods Approach with Focus Groups and Surveys | Adults Interested in Quit Smoking | Questionnaires and Focus Group Session Brainstorming | 40 Adults | USA |
| 54 | Davis et al (2016) “Taking mHealth Forward: Examining the Core Characteristics” | Literature Review | n.a. | Literature | 198 Sources | n.a. |
| 55 | Ose et al (2019): “Exploring the Potential for Use of Virtual Reality Technology in the Treatment of Severe Mental Illness Among Adults in Mid-Norway: Collaborative Research Between Clinicians and Researchers” | Focus Group Interviews | Researchers, the Manager at a District Psychiatric Center, and the Manager of the Local Municipal Mental Health Service | Collaborative Research Team | n.a. | Norway |

Source: Own Depiction
