# Supplementary material for: Potential Benefits and Risks Resulting From the Introduction of Health Apps and Wearables Into the German Statutory Health Care System: Scoping Review
Source: JMIR Mhealth Uhealth. 2020 Sep 23;8(9):e16444. doi: 10.2196/16444 (PMC7542416; doi:10.2196/16444)
Supplement: Multimedia Appendix 2 [file mhealth_v8i9e16444_app2.docx]

Multimedia Appendix 2. Study Aims and Findings

| # | Study | Aim | Findings |
| --- | --- | --- | --- |
| PubMed Search: Health Apps and Wearables n= 37, Eligible n= 20 | | | |
| 1 | Henriksen et al (2018): "Using Fitness Trackers and Smartwatches to Measure Physical Activity in Research" | - Analyzing change of consumer market for wearables - Giving an overview of devices | - 11 most relevant brands for wearables are Apple, Fitbit, Garmin, Mio, Misfit, Polar, PulseOn, Samsung, TomTom, Withings and Xiaomi, - Number of brands decreases over time - Number of wearables on the market increases with additional sensors |
| 2 | Mercer et al (2016): "Acceptance of commercially available wearable activity trackers among adults aged over 50 and with chronic illness" | - Analyzing the perceived acceptability of wearables to increase personal fitness | - mHealth has a significant potential to help older patients to become more active - Help to overcome barriers between patients and doctors |
| 3 | Firth & Torous (2015): "Smartphone Apps for Schizophrenia: A Systematic Review" | - Reviewing literature on apps for mental illnesses or schizophrenia | - People with schizophrenia are willing to record and engage with Apps to self-direct therapy - There seems to be a potential benefit in the use of apps for psychotic illnesses |
| 4 | Dimitrov (2016): "Medical Internet of Things and Big Data in Healthcare" | - Examining the potential health data collected through apps and wearables | - Apps will improve the communication between doctors and their patients - Storing the data collected by different sensors in a medical cloud and connecting the cloud to all relevant health institutions might have a significant cost reduction potential - Digital health advisory will play an important role in the future |
| 5 | Haghi et al (2017): "Wearable Devices in Medical Internet of Things: Scientific Research and Commercially Available Devices" | - Review about wearables, health apps and smart clothing in scientific papers and commercial websites | - Potential for the medical use of self-tracked health data might be significant - Sensors in the area of semiconductor technology have improved to be more precise |
| 6 | Vahabzadeh (2016): "Digital Suicide Prevention: Can Technology Become a Game-changer?" | - Review the technical possibilities and feasibility to monitor and observe psychological health data in apps to predict the risk of suicide | - Significant potential in the supervision of self-tracked psychological data to prevent suicide risk - Care should be taken because these technologies could be the source of error |
| 7 | Lobelo et al (2016): "The wild wild west: A Framework to integrate mHealth software applications and wearables to support physical activity assessment, counseling and interventions for Cardiovascular Disease risk reduction" | - Review the utility of implementing health apps in the existing health care system - Organizational framework to support the professional use of self-monitored data | - The use of mHealth to monitor and analysis health data seems promising in cardiovascular risk reduction - These apps also help to motivate users to conduct a different life style - The integration of self-monitored health data in the clinical care could be challenging |
| 8 | Wiesner et al (2018): "Technology Adaption, Motivational Aspects, and Privacy Concerns of Wearables in the German running community: Field Study" | - Analyzing the motivation and data privacy concerns of healthy and active people who use wearables to track their fitness. | - 73% of running community uses fitness tracking to monitor their exercises - 42 % of the participants are not concerned if their data would be shared with others - 32 % would share their data with their physician. |
| 9 | Urban (2017): "This really takes it out of you! The senses and emotions in digital health practices of the elderly" | - Analyzing how well mHealth is suited for the use of elderly people - What emotions does the use of mHealth trigger | - Elderly people develop negative emotions linked to aging stereotyped by using mHealth - Using mHealth also motivated to engage in more physical activity. |
| 10 | Ehn et al (2018): "Activity Monitors as support for older persons' physical activity in daily life: Qualitative Study of Users' Experience" | - Outlining the user experience of older people monitoring their health data with variables in daily life | - When monitoring health data with wearables, the physical activity of the probands increases - Indicates a high potential to encourage more physical activity for older people - Usability and reliability need to be improved to fit the consumer preferences of the group |
| 11 | Brandt et al (2018): "General Practitioners' Perspective on eHealth and lifestyle change: qualitative interview study" | - Outlining the practitioners' perspective on the use of self-tracked data for life style enhancement in Denmark | - General practitioners’ use mHealth applications constantly for themselves but rarely for their patients - The general practitioners assess the incorporation of self-tracked data in the eHealth system as beneficial |
| 12 | McCallum et al (2018): "Evaluating the Impact of Physical Activity Apps and Wearables: Interdisciplinary Review" | - To assess the research designs and methods of literature on engagement, acceptability and effectiveness research of physical activity apps. | - Most articles on PA apps are randomized controlled trials - A general evaluation guideline of health apps is urgently needed - In most cases, an optimization based on the evaluation of the app does not take place |
| 13 | Cresswell et al (2018): "Five key strategic priorities of integrating patient generated health data into United Kingdom electronic health records" | - Recommendation of five key strategies for the integration of self-monitored health data into the UK health system. | - Integration of self-monitored data into clinical applications is going to be a complex future process - The study has identified technology, user, legal, and political related factors as main inhibitors for progress |
| 14 | Paré et al (2018): "Diffusion of the Digital Health Self-Tracking Movement in Canada: Results of a National Survey" | - Analyzing the motivation of self-tracking and non-tracking - Outlining possible barriers to the technology and benefits | - 66% of the participants use on a regular basis one or more applications to track their health - There might be a high potential in home-monitoring health data of chronic ill patients with apps or wearables, increasing the patient empowerment. |
| 15 | Hicks et al (2019) “Best practices for analyzing large-scale health data from wearables and smartphone apps” | - To create an manual on how to use large-scale PGHD sets to understand the relationship between health behavior and environmental, social or personal factors | - The challenge about producing large-scale PGHD research is that the data sets are most of the times ‘messy’, important data is often incomplete or shows selection bias - The report give a manual on how to produce successful PGHD quantitative studies |
| 16 | Lüttke et al (2018): “E-Health in diagnosis and therapy of mental disorders. Will therapists soon become superfluous?” | - To get an overview about the state of research on mental disease mHealth solutions, application possibilities, products and there effectivity in the fields of diagnostics and therapy. | - Using mHealth apps complementary to traditional methods to diagnose mental disease seems promising and effective - There is great potential to reduce costs in the preventive care of mental disease and allocate personnel resources more effectively - Screening and monitoring through Apps and wearables reduces barriers and could potentially improve the therapist-patient relationship |
| 17 | Turankhia & Kaiser (2016): “Transforming the care of atrial fibrillation with mobile health” | - To summarize the limitations and data gaps of monitoring and treating AF patients | - Especially wearables seem promising in the application of symptoms, heart rates and rhythms surveillance of AF or AF risk patients - Reimbursement models should encourage AF patients to self-track and share their data - These technologies have the potential to decrease the screening costs and enhance early detection of AF |
| 18 | Heintzman (2016). “A Digital Ecosystem of Diabetes Data and Technology: Services, Systems, and Tools Enabled by Wearables, Sensors, and Apps” | - To get an overview about the technological possibilities and effectivity of mHealth solution to accompany diabetes treatment | - PGHD in diabetes research has great potential to further research and understand glycemia in a realistic setting - mHealth enables individualized guidance for patients and potentially decreases costs because of less professional surveillance and guidance |
| 19 | Knight & Bidargaddi (2018): ”Commonly available activity tracker apps and wearables as a mental health outcome indicator: A prospective observational cohort study among young adults with psychological distress” | - To explore the potential of health apps and wearables as valid mental health assessment tool | - The collection of PGHD and the self-management of mental disease through apps might contribute to the patient empowerment - The collection and processing of PGHD helps clinicians to improve clinical care through a better understanding of behavioral indicators |
| 20 | Ramkumar et al (2017): “Open mHealth Architecture: A Primer for Tomorrow’s Orthopedic Surgeon and Introduction to its use in Lower Extremity Arthroplasty” | - To get an general overview about the open mHealth architecture and its possibilities within orthopedic surgery | - Post-surgery interactive apps might help to manage pain and leads to patient empowerment - Apps might lead to a more efficient allocation of resources |
| Google Scholar Search: "Health Apps (and) Wearables" n= 36, Eligible n= 16, n= 4 Purposeful Sampled Articles in German | | | |
| 21 | Canhoto & Arp (2017): "Exploring the factors that support adoption and sustained use of health and fitness wearables" | - To outline the key factors why people use mHealth applications and the key stakeholders interested in the health data | - The current German wearable user is in average young and sportive - The users were especially interested in fitness apps or wearables, if they were designed to be goal oriented - The visualization of the data and the possibility for data competition are significant variables |
| 22 | Albrecht (2016): "Gesundheits-Apps und Prävention" | - Reviewing the general conditions for using health apps in Germany | - Most Apps are clustered as Lifestyle apps - There is little or no academic research on wearables - The number of existing apps seems problematic because the software is in most cases not compatible and data is not interchangeable |
| 23 | Groß & Schmidt (2018): "Health und Gesundheitsapps aus medizinischer Sicht" | - Discussing the ethical assessment of health apps | - The philosophical evaluation models for health apps benefit the users and the doctors to categorize them and to understand their chances and risks |
| 24 | Genes et al (2018): "From smartphone to EHR: a case report on integrating patient-generated health data" | - To evaluate the aspects of self-monitored data integration into the practitioners daily routine for disease management | - The potential of integrating and sharing self-monitored data with the physician is considered high for the patients - There might be some pitfalls with the incorporation of the data in the daily routine of the physician. |
| 25 | GfK (2016): "Global GfK survey: Health and Fitness Tracking" | - Outlining the market for wearables and health tracking in a comparative setting | - 33% of the people in the 16 countries use health tracking - The countries with most consumers using health tracking are China, Brazil and the USA - Most people use health tracking to improve or maintain the personal status of physical fitness. |
| 26 | Krebs & Duncan (2015): "Health App Use Among US Mobile Phone Owners: A National Survey" | - To analyze the sociodemographic variables, motivation and history of health app und wearable users | - There is still a large proportion of citizens who do not use health apps or when using, already stopped using them - Health apps should better consider consumer concerns and their usability needs |
| 27 | Becker et al (2014): "mHealth 2.0: Experiences, Possibilities, and Perspectives" | - Analyzing the potential of health apps from a psychological, legal, clinical and technological perspective | - The potential of health apps is high - Apps struggle with their "trustworthiness" - Apps are in general underutilized - When designing new Health apps, all stakeholders should be involved |
| 28 | Huckvale et al (2015): "Unaddressed privacy risks in accredited health and wellness apps: a cross-sectional systematic assessment" | - To outline the data protection gaps of certified "clinically safe" mHealth applications | - 89% of the Health apps uploaded information to online services - 66% of the apps did not encrypt information when sending - 92% did not encrypt information when stored locally - There are significant gaps in the compliance with data protection principles, assuming that certification programs might not deliver the desired outcome |
| 29 | Urrea et al (2015): "Mobile Health Initiatives to improve outcomes in primary prevention of cardiovascular disease" | - To outline the potential Health apps in preventive care for cardiovascular disease | - There are many health applications in this segment available - Effectivity remains insufficiently studied - Important to develop a guide for the evaluation of effectivity |
| 30 | Statista (2018): "Mobile Health" | - To describe characteristics of wearable users in Germany | - Wearable users are interested in the themes health, fitness and nutrition and want to use the apps for self-optimization - the youngest group (18-29 years old) holds most health app users - 55% would share their self-tracked health data in any case with their doctor, 15% with their health insurance and 7% would share with an internet company |
| 31 | Park et al (2018): "Why do young people use fitness apps? Cognitive characteristics and App quality" | - To outline the motivation or reasons why people use health apps | - Young college students use health apps because of health concerns, innovation, expectation and self-efficiency |
| 32 | Schoeppe et al (2017): "Apps to improve diet, physical activity and sedentary behavior in children and adolescents: a review of quality, features and behavior change techniques" | - To evaluate health apps available for children on a five point mobile rating scale | - Popular apps for children to improve physical activity, diet and sedentary behavior have a moderate quality - It is important to promote the engagement of the user while using the app and they should respond better to the individual user’s needs |
| 33 | Wichmann et al (2018): "Apps for physical activity promotion. Attitudes, acceptance and utilization preferences among adults aged 50 years and above: results of focus group discussions" | - To gain insights into the motivation of health app use among a focus group aged 50 years and older | - Users of health apps rated the use of health Apps higher than the non-user focus group - People had concerns about data safety and disliked apps, in which data had to be entered manually - Simple apps with only a few features were liked more than apps with many features |
| 34 | Cristóvao Veríssimo (2018): "Usage intensity of mobile medical apps: A tale of two methods" | - Analyzing impact factors like peer influence, perceived usefulness and perceived usefulness of use | - High health app usage among the doctors and medical students depends on perceived usefulness and ease of use - Apps, which are used to support the clinical practice attract an increasing number of doctors - Senior doctors and young female doctors tend to use less health apps, if there is little peer influence, little perceived usefulness and little ease of use |
| 35 | Peng et al (2016): "A qualitative study of user perceptions of mobile health apps" | - To outline the opportunities and challenges of health applications | - 57% of the participants used health apps before - The main reasons for non-usage was lack of awareness, lack of app literacy, cost and the lack to need a health app - The reasons for people to use health apps are motivation, dedication, social competition, entertainment and a reward system |
| 36 | Somers et al (2019): "Valuing Mobile Health: An Open-Ended Contingent Valuation Survey of a National Digital Health Program" | - To evaluate the consumer perspective on potential benefits of mHealth | - Consumers appreciate health apps, which promote well-being, health care control and social connectivity - Health apps should be tailored to the needs of the consumers and potential barriers should be better understood - There is a positive correlation between willingness to pay of health apps and younger age and income - People who are in better health shape use mHealth more frequently |
| 37 | Chung et al (2016): "Harnessing person-generated health data to accelerate patient-centered outcomes research: the Crohn’s and ColitisFoundation of America PCORnet Patient Powered Research Network" | - To establish an online platform IBD patients to Upload PGHD and to enhance a patient centered research network | - The platform is popular amongst the IBD patients, about 14 200 patients registered online and chose which data they want to upload and how actively they want to participate in the formulation of research questions - The platform as a network and data-base helps participants to gain insights about their condition and facilitates the monitoring and management of the disease |
| 38 | Petersen et al (2019): “The shifting politics of patient activism: From bio-sociality to bio-digital citizenship” | - To analyze the shifting dynamics and media activity of HIV/AIDS and breast cancer groups | - Bio-based identity is an ever growing concept, enhanced through the internet and new communication and networking channels - People gain more scientific and personal information such as reports based experiences through online health platforms - Collected health data is seen as valuable in the future but there is a current asymmetry between those who collect and those who are selling the data |
| 39 | Armstrong (2016): “What happens to data gathered by health and wellness apps?” | - To report the processing of self-tracked health data | - Most apps or wearables offer little or no data protection for their consumers even though their use is encouraged by the NHS - There is a current lack of data privacy education because many consumer agree to terms and conditions without reading them - Many apps and wearables sell personal fitness data without even encrypting them - There is a need for more government regulations about PGHD processing |
| 40 | Montgomery et al (2018): “Health Wearables: Ensuring Fairness, Preventing Discrimination, and promoting Equity in an Emerging Internet-of-Things Environment” | - To outline data privacy concerns when using big data to analyze large-scale PGHD | - Government regulations are needed to enhance fairness and equity when for example health insurance companies use and analyze PGHD - Further data privacy education is needed to encourage the companies to share their profit from data selling with the consumers - Transparency about data processing is one of the most important points to gain the trust of the consumer |

| JMIR Publications Search: "Health Apps AND Wearables" n= 59 , Eligible n= 15 |
| --- |

| 41 | Hartmann et al (2019): “Utilization of Patient-Generated Data Collected Through Mobile Devices: Insights From a Survey on Attitudes Toward Mobile Self-Monitoring and Self-Management Apps for Depression” | - To understand the usage of self-monitoring via health apps and wearables of depression patients | - Apps may decrease treatment challenges for depression patients - Sharing the self-tracked data with therapist might improve communication and increase adherence |
| --- | --- | --- | --- |
| 42 | Mosconi et al (2019): “Use of Health Apps and Wearable Devices: Survey Among Italian Associations for Patient Advocacy” | - To research health care advocacy associations for patients about their opinion of health apps | - Many patients already use health apps - Accessibility of apps should improve - Many patients already appreciate use of apps |
| 43 | Ernsting et al (2019): “Associations of Health App Use and Perceived Effectiveness in People With Cardiovascular Diseases and Diabetes: Population-Based Survey” | - To research perceived usefulness of health apps for managing diabetes and CVD | - Younger study participants rated health apps more effective than older people - Health knowledge is an important factor as well as technology literacy - Usability of apps should be increased |
| 44 | Gabriels & Moerenhout (2018): “Exploring Entertainment Medicine and Professionalization of Self-Care: Interview Study Among Doctors on the Potential Effects of Digital Self-Tracking” | - To understand doctors’ opinion about patient self-tracking | - Opportunity for more personalized care - Professionalization of self-care - Patient empowerment - But self-care also as hard work |
| 45 | Tabi et al (2019): “Mobile Apps for Medication Management: Review and Analysis” | - To create an overview about medication plan apps | - There is a need for a professional health app platform - Patient empowerment through health apps - Too many apps on market - Non-transparent apps, need to regulate standards |
| 46 | Jamaladin et al (2018): “Mobile Apps for Blood Pressure Monitoring: Systematic Search in App Stores and Content Analysis” | - To evaluate blood pressure monitoring apps | - Quality of many apps poor - Too many apps on market - Few apps have a very good quality, these enhance patient self-management |
| 47 | Christmann et al (2017): “Stress Management Apps With Regard to Emotion-Focused Coping and Behavior Change Techniques: A Content Analysis” | - To give an overview about stress-management apps | - Apps include conventional methods of behavior change methods and new methods of emotion focused stress management - Many apps combine different stress management strategies - Evidenced-based content missing in health app |
| 48 | Collado-Borrell et al (2018): “Oncology Patient Interest in the Use of New Technologies to Manage Their Disease: Cross-Sectional Survey” | - To understand the patients’ needs and willingness of disease self-management | - Patients very interested in apps for self-management - Healthcare providers should play a more active role in the recommendation of a health app and supervision of its use |
| 49 | Morrow Lipschitz et al (2019): “Adoption of Mobile Apps for Depression and Anxiety: Cross-Sectional Survey Study on Patient Interest and Barriers to Engagement” | - To evaluate the mental health app usage of patients | - Patients are generally interested in using these technologies but do not use them yet - Privacy concerns are addressed - Health care providers should supervise app usage |
| 50 | Martinez-Millana et al (2018): “App Features for Type 1 Diabetes Support and Patient Empowerment: Systematic Literature Review and Benchmark Comparison” | - To evaluate features of diabetes apps | - Apps have potential to educate patients, track diabetes data and share the data with important health care stakeholder - Many apps just enable data collection - Apps should include more individualized self-management features |
| 51 | Mackert et al (2016): “Health Literacy and Health Information Technology Adoption: The Potential for a New Digital Divide” | - To research the impact of health literacy on the use of health apps and wearables | - Health literacy is significantly associated with nutrition and fitness app usage - People whore are more health literate find the use of health apps easier |
| 52 | Hoffmann et al (2017): “Gamification in Stress Management Apps: A Critical App Review | - To give an overview about the use of gamification for stress management apps | - App users use little or no gamification in stress management apps - Benefits of gamification should be more exploited in health apps to enhance adherence and behavior change |
| 53 | Hartzler et al (2016): “Prioritizing the mHealth Design Space: A Mixed-Methods Analysis of Smokers’ Perspectives” | - To give an overview about preferred features of quit smoking apps for smokers | - User preferences and best practice treatments should be included in the health app design - People prefer rather personalized and interactive tools and features - App should be designed more evidence based |
| 54 | Davis et al (2016) “Taking mHealth Forward: Examining the Core Characteristics” | - To give an overview about all core characteristics of mobile health apps | - People are already using technologies and the adapt to the usage quite fast - Connectivity is an important factor about the usage of health apps - High potential for self-management - Evidence based treatments should be included |
| 55 | Ose et al (2019): “Exploring the Potential for Use of Virtual Reality Technology in the Treatment of Severe Mental Illness Among Adults in Mid-Norway: Collaborative Research Between Clinicians and Researchers” | - To research the potential of VR as complimentary mental illness treatment | - VR has great potential in the treatment of mentally ill patients - Greatest potential lies in social skills and countering self-isolation - Potential to educate family and friends of patient about the mental disease |

1. The user group of health apps and wearables
2. Effectivity of health apps and wearables to improve health
3. The potential of brought along self-tracked data
4. Concerns and data privacy risks

Source: Own Depiction.
